# Memory-Like Antigen-Specific Human NK Cells from TB Pleural Fluids Produced IL-22 in Response to IL-15 or *Mycobacterium tuberculosis* Antigens

**DOI:** 10.1371/journal.pone.0151721

**Published:** 2016-03-31

**Authors:** Xiaoying Fu, Sifei Yu, Binyan Yang, Suihua Lao, Baiqing Li, Changyou Wu

**Affiliations:** 1 Institute of Immunology, Zhongshan School of Medicine, Key Laboratory of Tropical Disease Control Research of Ministry of Education, Sun Yat-sen University, Guangzhou, China; 2 Chest Hospital of Guangzhou, Guangzhou, P. R. of China; 3 Department of Immunology, Research Center of Immunology, Bengbu Medical College, Bengbu, P. R. of China; Karolinska Institutet, SWEDEN

## Abstract

Our previous result indicated that memory-like human natural killer (NK) cells from TB pleural fluid cells (PFCs) produced large amounts of IFN-γ in response to *Bacille Calmette Guerin* (BCG). Furthermore, recent studies have shown that human lymphoid tissues harbored a unique NK cell subset that specialized in production of interleukin (IL)-22, a proinflammatory cytokine that mediates host defense against pathogens. Yet little information was available with regard to the properties of IL-22 production by memory-like human NK cells. In the present study, we found that cytokines IL-15 induced and IL-12 enhanced the levels of IL-22 by NK cells from TB PFCs. In addition, IL-22 but not IL-17 was produced by NK cells from PFCs in response to BCG and *M*.*tb*-related Ags. More importantly, the subset of specific IL-22-producing NK cells were distinct from IFN-γ-producing NK cells in PFCs. CD45RO^+^ or CD45RO^-^ NK cells were sorted, co-cultured with autologous monocytes and stimulated with BCG for the production of IL-22. The result demonstrated that CD45RO^+^ but not CD45RO^-^ NK cells produced significantly higher level of IL-22. Anti-IL-12Rβ1 mAbs (2B10) partially inhibit the expression of IL-22 by NK cells under the culture with BCG. Consistently, BCG specific IL-22-producing NK cells from PFCs expressed CD45RO^high^NKG2D^high^granzyme B^high^. In conclusion, our data demonstrated that memory-like antigen-specific CD45RO^+^ NK cells might participate in the recall immune response for *M*. *tb* infection via producing IL-22, which display a critical role to fight against *M*. *tb*.

## Introduction

Tuberculosis (TB) remains a leading cause of mortality worldwide [1]. Additionally, the risk of developing active tuberculosis was aggravated by a variety of insufficient prevention efforts, such as HIV infection, diabetes, drug-resistant strains of *mycobacterium tuberculosis* and immunosuppressant treatment [2–4]. Tuberculous pleurisy is the second most frequent manifestation of extra-pulmonary tuberculosis after TB infection in lymph node that leads to the accumulation of protein-enriched fluids and the recruitment of specific inflammatory lymphocytes into the pleural space. Therefore, tuberculous pleurisy is a good model for the study of TB specific cells [5,6].

Both innate and adaptive immune systems contribute to host defense against infection with *M*. *tb* [7–13]. Human natural killer (NK) cells have been dissected into CD56^dim^ and CD56^bright^ subpopulations possessing either lytic or cytokine production, which are believed to display an important role in innate immunity to microbial pathogens [14,15]. It has been reported that NK cells are potent producer of IFN-γ and associated with early resistance against *M*. *tb* infection [16,17]. Moreover, recent studies have found that human NK cells produce not only IFN-γ but also IL-22, which display an important role in host defense and homeostasis, and are critical for induction of antimicrobial peptides in response to bacterial infections [18]. IL-22 is a member of the IL-10 cytokine family that is produced by special immune cell populations including CD4^+^ and CD8^+^ T cells, which display either a protective or a pathogenic role in chronic inflammatory diseases [19–23]. NK-IL-22 cells provide an innate source of IL-22 that may help constrain inflammation and protect mucosal sites [18,24].

Traditionally, immunological memory has been regarded as a unique feature of the adaptive immune response and mediated in an antigen-specific manner by T and B lymphocytes [25]. However, recent studies on NK cells are challenging the concept of immunological memory [26]. Scientists have identified that mouse NK cells exhibit memory-like properties, defined by an initial activation event, a subsequent return to the resting state and followed by enhanced IFN-γ production upon re-stimulation [27]. Another group investigated both on human and murine NK cells that initial stimulation with the cytokines, IL-12, IL-15 plus IL-18, results in the majority of NK cells producing IFN-γ, and after 1 to 3 weeks these cells exhibit memory-like NK properties, with increased IFN-γ production following re-stimulation with cytokines or via the engagement of activating NK cell receptors [28,29]. In addition, study on mouse NK cells demonstrated that a subset of NK cells in the liver acquired antigen-specific memory to various haptens and viruses [30]. Tian and colleges investigated that a subpopulation of murine CD49a^+^DX5^-^ NK cells resided in liver possessed memory potential and conferred hapten-specific CHS responses upon hapten challenge [31]. Collectively, these findings demonstrated that memory-like NK cells are long-lived and exhibit a recall response.

In the previous study, our data demonstrated that memory-like human CD45RO^+^ NK cell were migrated to tuberculous pleural fluid via the IP-10/CXCR3 and SDF-1/CXCR4 axis, which produced more IFN-γ than CD45RO^-^ NK cells from PFCs in response to BCG [17, 32]. In the current study, we further evaluated the cytokine secretion by memory-like NK cells from PFCs. Our results illustrated that IL-15 and IL-12 had different effects on the production of IFN-γ and IL-22 by NK cells both from PFCs and PBMCs. More importantly, IL-22 was produced by NK cells from PFCs under the stimulation with BCG and *M*.*tb* related Ags. In addition, sorted memory-like CD45RO^+^ NK cells from PFCs produced significantly higher level of IL-22 in response to BCG compared with CD45RO^-^ NK cells, which was dependent on IL-15 but not IL-12. Taken together, our data demonstrated that memory-like human CD45RO^+^ NK cells might participate in the recall immune response to *M*. *tb* infection via producing IL-22, which provide a novel light in the biological function and NK cell-based immunotherapy.

## Material and Methods

### Study participants

Twenty-eight patients with tuberculous pleurisy (11 females and 17 males, ranged from 19 to 66 years of age) were recruited from the Chest Hospital of Guangzhou, China. Diagnosis of pleural effusion from TB etiology was based on one of the following criteria: (1) demonstration of MTB on pleural fluid smear (by the Ziehl-Neelsen method); (2) pleural fluid or pleural biopsy specimens growing *M*. *tuberculosis* on Lowenstein-Jensen medium; or (3) histological evidence of caseating granuloma on biopsy specimens of pleural tissue with positive staining for *m*. *tuberculosis*. Twenty-eight healthy donors (12 females and 16 males, ranged from 21 to 58 years of age) were recruited from Zhongshan School of Medicine. Both patients and healthy donors who had been diagnosed with HIV, hepatitis B virus (HBV), hepatitis C virus (HCV), or with a history of autoimmune diseases were excluded from this study.

Written informed consent was obtained from all patients and healthy donors. Ethics approval for the present study was obtained from the ethics committee of the Zhongshan School of Medicine, Sun Yat-sen University (Guangzhou, China) and the Chest Hospital of Guangzhou (Guangzhou, China).

### Reagents and mAbs

Freeze-dried BCG (Chengdu Institute of Biological Products, Chengdu, China) was reconsitituted in a 0.9% sodium chloride solution to a concentration of 1 mg/mL prior to use. Sonicatd *M*.*tb* protein antigen (TB-Ag) was generously provided by Prof. Baiqing Li (Department of Immunology, Bangbu Medical College, Anhui Key Laboratory of Infection and Immunity, Bangbu, China). The following mAbs were used for phenotypic and intracellular cytokine analysis: phycoerythrin (PE)-labeled anti-CD3, anti-CD14, anti-CD56, anti-CD45RA, anti-IL-17, fluorescein isothiocyanate (FITC)-labeled anti-CD3, anti-CD45RA, anti-CD45RO, anti-granzyme B, Phycoerythrin-Cy7 (PE-cy7)-labeled anti-CD69, anti-CD56, allophycocyanin (APC)-labeled anti-CD3, anti-IFN-γ anti-IL-22 were obtained from BD Pharmingen (San Jose, CA, USA). FITC-labeled anti-CD16 was purchased from Biolegend (CA, USA). PE-labeled anti-NKG2D were obtained from R&D Systems (MN, USA), respectively.

### Preparation of PFCs and PBMCs

TB pleural fluid (PF) was collected by therapeutic thoracentesis from tuberculous patients. TB pleural fluid cells (PFCs) and peripheral blood mononuclear cells (PBMCs) were isolated by Ficoll-Hypaque (Tianjin Hao Yang Biological Manufacture, Tianjin, China) gradient centrifugation within 24 h of sampling and washed twice in Hanks’ balanced salt solution. The cells were suspended at a final concentration of 2×10^6^/mL in complete RPMI-1640 medium (Invitrogen, Grand Island, NY) supplemented with 10% heat-inactivated fetal calf serum (FCS; HyClone, Logan, UT), 100U/mL penicillin, 100μg/mL streptomycin, 2mM L-glutamine and 50μM 2-mercaptoethanol.

### Flow Cytometry

The cells were washed twice with PBS buffer containing 0.1% BSA and 0.05% sodium azide (FACS buffer). For surface staining, cells were incubated with the respective mAbs at 4°C for 30 min in dark, then washed twice and fixed in 0.5% paraformaldehyde before acquisition. Cells were gated on CD3^+^ cells and NK cells were divided into three subsets of CD56^high^CD16^-^, CD56^low^CD16^-^ and CD56^+^CD16^+^ based on the expression of cell surface CD56 and CD16 molecules. For the detection of intracellular cytokines, the cells were incubated at a concentration of 2×10^6^/mL with 20 μg/mL BCG for 8 h in the presence of berfeldin A (BFA, 10μg/mL; Sigma-Aldrich, St Louis, MO). Intracellular staining for Granzyme B was performed after fixation in 4% paraformaldehyde, followed by permeabilization and staining in FACS buffer containing 0.1% saponin. These cells were collected by FACSCalibur^™^ (Becton Dickinson, San Jose, CA) or FACSAria II (BD company, San Jose, CA, USA) and analyzed with FlowJo software (Treestar, San Carlos, CA).

### Isolation of cell subsets

NK cells were obtained by negative selection from PFCs and PBMCs using the NK cell Isolation KitII and CD14^+^ cells were isolated from PFCs by positive selection with anti-CD14 microbeads (Miltenyi Biotec, Bergisch Gladbach, Germany) according to the manufacturer’s protocol. For isolation of CD45RO^+^ and CD45RO^-^ NK cells, purified NK cells from PFCs were stained with FITC-labeled anti-CD45RO for 30 min at 4°C in dark and washed twice with the buffer as described above. The cells were suspended in the buffer and sorted by FACS AriaII flow cytometer for the subsets of CD45RO^+^ or CD45RO^-^ NK cells from PFCs. The purity of cells assessed by flow cytometry exceeded 95% for each cell subset.

### Cell culture condition

PFCs or PBMCs were incubated at a concentration of 2×10^6^/mL with different doses of rhIL-12 (eBioscience San Diego, CA, USA) or rhIL-15 (Peprotech, Rocky Hill, NJ, USA), and cell-free supernatants were collected at different time points and assessed by ELISA for IFN-γ (BD Bioscience Pharmingen) and IL-22 (R&D System) production according to the manufacturer’s protocols, respectively. For the detection of intracellular cytokines, the cells were cultured for 8 h in the presence of berfeldin A (BFA, 10μg/mL; Sigma-Aldrich, St Louis, MO, USA) and then subjected to flow cytometry analysis. PFCs or PBMCs were incubated at a concentration of 2×10^6^/mL with BCG (20 μg/mL, Chengdu Institute of Biological Products, Chengdu, China) or *M*. *tb*-Ag for 8 h in the presence of BFA for the detection of IFN-γ, IL-22 and IL-17 by flow cytometry. Sorted CD45RO^+^ or CD45RO^-^ NK cells were treated with or without BCG for 48 h. Sorted CD45RO^+^ or CD45RO^-^ NK cells were co-cultured with purified CD14^+^ cells (at ratio of 4: 1) in the presence or absence of BCG, BCG plus 2B10 (anti-IL-12Rβ1 mAbs, 10μg/mL, Hoffmann-La Roche Inc., USA), LPS (100ng/mL, Sigma-Aldrich, St Louis, MO, USA) and LPS plus 2B10 for 48 h. Cell-free supernatants were harvested and assessed by ELISA for the production of IFN-γ and IL-22 according to the manufacturer’s protocols, respectively.

### Statistical analysis

Data were reported in terms of medians, minimum and maximum values or as mean±SEM. Comparison between two groups was performed by unpaired student’s t-tests. A value of P<0.05 was considered significant.

## Results

### IL-15 or/and IL-12 in a dose- and time-dependent manner induced the production of IL-22 from PBMCs

**Fig 1:**
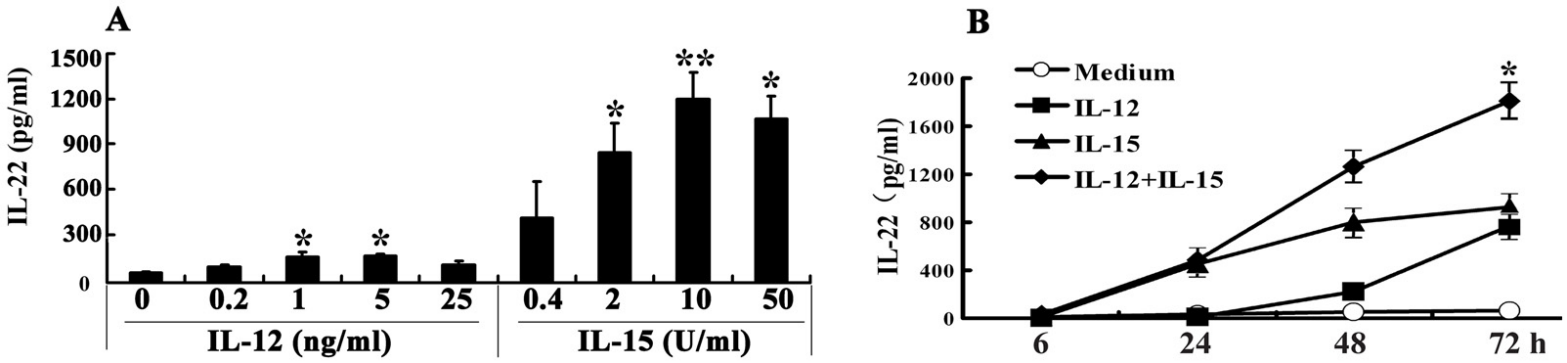
IL-15 and IL-12 differently induced the production of IL-22 in a dose- and time-dependent manner by human PBMCs. PBMCs were incubated with or without IL-12 or IL-15 in different doses. The concentrations of IL-22 (A) in the culture supernatants were determined by ELISA. Mean values of IL-22 (n = 5) were shown as Mean±SD. In addition, PBMCs were incubated with or without IL-12 or IL-15 or IL-12 plus IL-15. The cells were harvested at different time points (B). The concentrations of IL-22 (A) in the culture supernatants were determined by ELISA. Mean values of IL-22 (n = 5) were shown as Mean±SD. Student’s t-test was used for statistical analysis, *P<0.05 and **P<0.01.

To detect whether IL-15 or IL-12 had any effect on the production of IL-22 from PBMCs, we stimulated PBMCs 48–72 h with various concentrations of IL-15 or IL-12 and the levels of IL-22 in the cell-free culture supernatants were assessed by ELISA. The results showed that without any stimulation, PBMCs did not produce IL-22. When IL-12 was added into cell cultures, a small amount of IL-22 was induced from PBMCs. Simultaneously, addition of IL-15 to cell cultures markedly induced the production of IL-22 in a dose-dependent manner (Fig 1A). The optimal concentration of IL-15 and IL-12 for the induction of IL-22 was 10U/mL and 5ng/mL respectively. As illustrated in Fig 1B, the production of IL-22 from PBMCs was induced by IL-15 in a time dependent manner. More interestingly, IL-15 plus IL-12 displayed a synergistic effect on the induction of IL-22 from PBMCs. Our previous studies had found that IL-15 and IL-12 differently induce the production of IFN-γ in a dose- and time-dependent manner by human PBMCs (S1 Fig).

### IL-15 and IL-12 induced the expression of IL-22 and IFN-γ by NK cells from PBMCs

**Fig 2:**
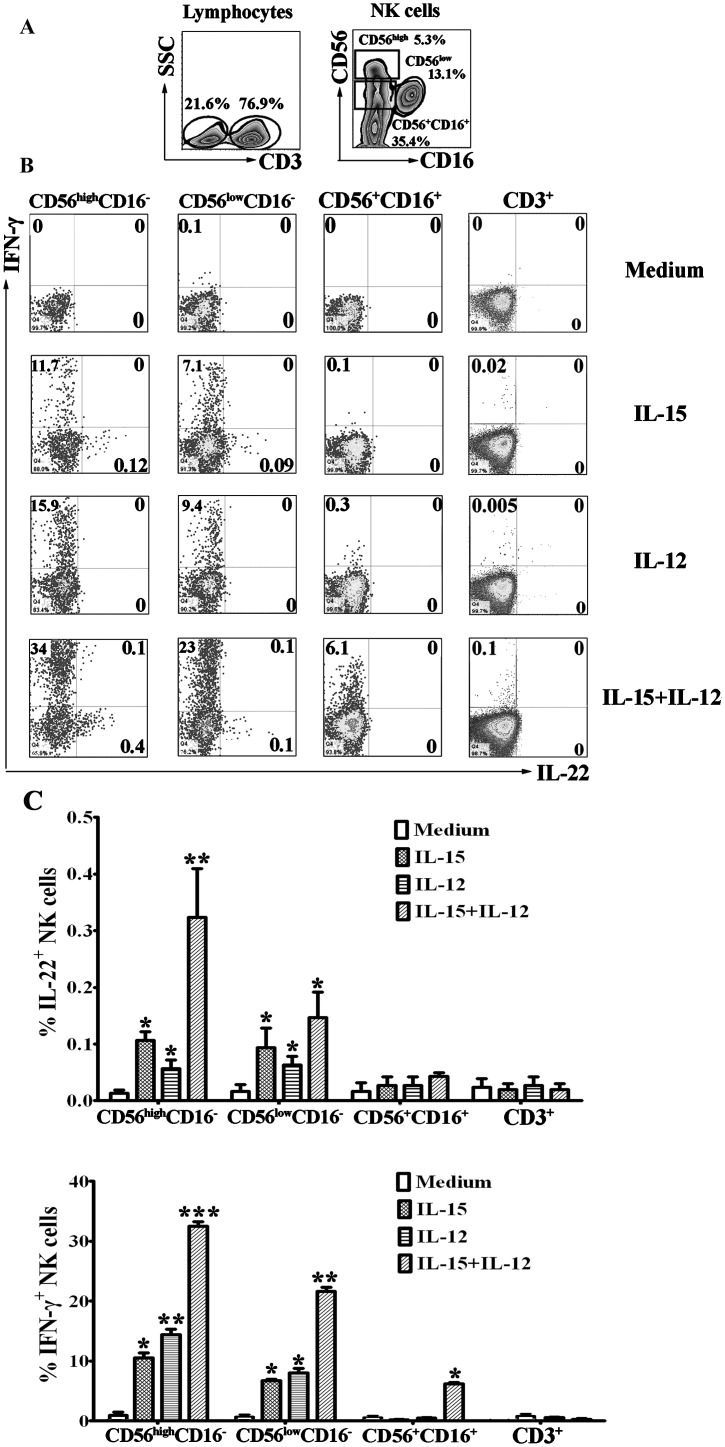
IL-15 and IL-12 induced the expression of IL-22 and IFN-γ by different subsets of NK cells from PBMCs. PBMCs were cultured with or without IL-12 or IL-15 or IL-12 plus IL-15 for 24 h in the presence of BFA. The cells were harvested, gated on lymphocytes and then gated on CD3^-^ and CD3^+^ cells. NK cells from CD3^-^ cells were divided into three subpopulations of CD56^high^CD16^-^, CD56^low^CD16^-^ and CD56^+^CD16^+^ cells (A). The expression of IFN-γ and IL-22 on three subsets of NK cells and CD3^+^ T cells were analyzed by FCM (B). Dot plots showed representative results with three separate experiments. (C). Statistical results were shown as mean±SD in histogram and error bars represent triplicates within the similar experiment. *P<0.05, **P<0.01 and ***P<0.001.

We further evaluated the subpopulations of IL-22-producing cells. PBMCs were stimulated in vitro with or without IL-15, IL-12 or IL-15 plus IL-12 in the presence of 10μg/mL brefeldin A. After stimulation for 8 h, the cell-surface markers and intracellular cytokines were stained with anti-CD3, anti-CD56, anti-CD16, anti-IL-22, anti-IFN-γ and isotype-matched controls. For flow cytometric analysis, the cells were gated on CD3^-^ and CD3^+^ cells. NK cells in CD3^-^ cells were divided into three subpopulations of CD56^high^CD16^-^, CD56^low^CD16^-^ and CD56^+^CD16^+^ NK cells, and the expression of IL-22 and IFN-γ were analyzed (Fig 2). Similarly with the previous study, the expression of IFN-γ in response to IL-12 was largely induced by NK cells but not CD3^+^ T cells. More importantly, the results demonstrated that the expression of IL-22 in response to IL-15 or IL-15 plus IL-12 was primarily by NK cells, especially by the subpopulation of CD56^high^CD16^-^ NK cells and CD56^low^CD16^-^ but not by CD3^+^ T cells.

### NK cells from PFCs and PBMCs were induced to the expression and production of IL-22 under the cultures with IL-15 or IL-15 plus IL-12

**Fig 3:**
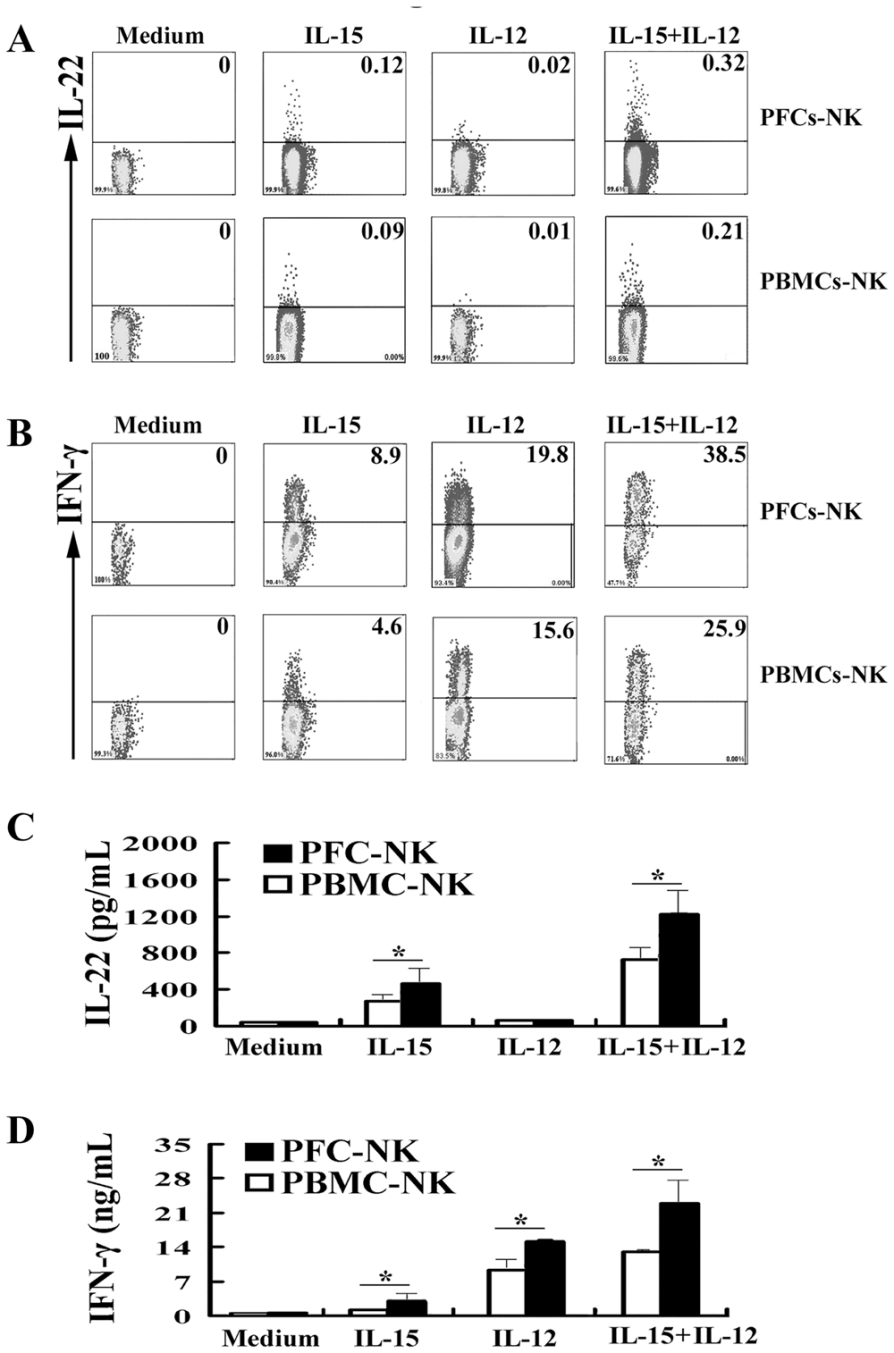
IL-15, IL-12 or IL-15 plus IL-12 induced NK cells from PFCs and PBMCs to expresse and produce IL-22 and IFN-γ. PFCs and PBMCs were stimulated with or without IL-15, IL-12 or IL-15 plus IL-12 for 24 h. The expression of IL-22 (A) and IFN-γ (B) from CD3^-^CD56^+^ NK cells was detected by FCM. Representative dot plots of five independent experiments were shown with similar results. Purified NK cells from PFCs and PBMCs were cultured with or without IL-15, IL-12 or IL-15 puls IL-12 for 24 h. The concentration of IL-22 (C) and IFN-γ (D) was detected by ELISA. Statistical results were shown as mean±SD in histogram and error bars represent triplicates within the similar experiment. *P<0.05.

Our previous study illustrated that NK cells from pleural fluid cells (PFCs) displayed an optimal role on the production of IFN-γ. To evaluate whether NK cells from PFCs induced the expression and production of IL-22 in response to IL-15 or IL-12. PFCs and PBMCs were stimulated with or without IL-15, IL-12 or IL-15 plus IL-12 for 24 h and the cells were harvested for the cytokine evaluation by intracellular cytokine stainings. As illustrated in Fig 3A and 3B, NK cells were gated on CD3^-^CD56^+^ cells and the expression of IL-22 and IFN-γ was analyzed. The results showed that NK cells from PFC expressed significantly higher levels of IL-22 compared with NK cells from PBMCs (0.15%±0.06% vs 0.06%±0.009%) in response to IL-15 but not IL-12. More importantly, IL-12 enhancd the expression of IL-22 under the stimulation with IL-15 (0.35%±0.007% vs 0.19%±0.003%). Similarly, NK cells from PFCs produced larger amount of IFN-γ than did NK cells from PBMCs in response to IL-12 (18.7%±2.3% vs 14.6%±2.5%). To confirm our results, NK cells were sorted by flow cytometry from PFCs and PBMCs, and sorted NK cells were cultured with or without IL-15, IL-12 or IL-15 plus IL-12. As illustrated in Fig 3C and 3D, sorted NK cells from PFCs produced higher levels of IL-22 and IFN-γ in response to IL-15 or IL-15 plus IL-12 compared to NK cells form PBMCs.

### IL-22 but not IL-17 was induced by NK cells from PFCs and PBMCs under the stimulation with BCG or *M*.*tb* related Ags

**Fig 4:**
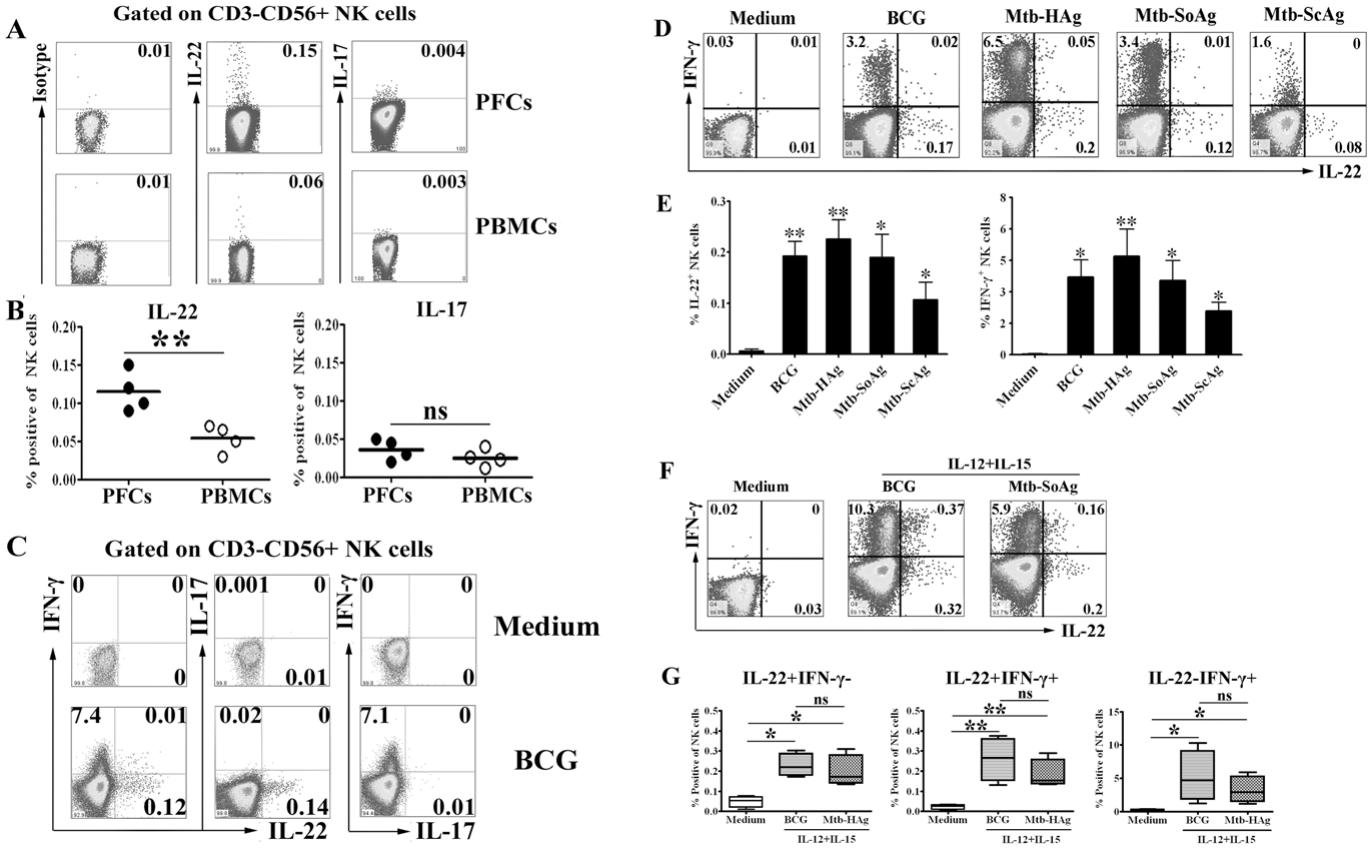
BCG and *M*.*tb*-related Ags induced more production of IL-22 and IFN-γ but not IL-17 by NK cells from PFCs but not PBMCs. (A) PFCs and PBMCs were stimulated with or without BCG. The expression of IL-22 and IL-17 by CD3^-^CD56^+^ NK cells was evaluated by FCM. Representative dot plots of five independent experiments were shown with similar results. (B) Statistical results of IL-22 and IL-17 expression by by CD3^-^CD56^+^ NK cells from PFCs and PBMCs. The median and individual frequencies for each patient and healthy volunteer were performed. (C) Analysis for the relationship of IL-22, IFN-γ and IL-17 expression by FCM. The dot plots were representative of four separate experiments with similar results. (D) PFCs were incubated with BCG and *M*.*tb*-related Ags for 8 h in the presence of BFA. The expression of IL-22 and IFN-γ by CD3^-^CD56^+^ cells were evaluated by FCM. The dot plots were representative of four separate experiments with similar results. (E) The statistical histograms were shown as mean±SD and error bars represent triplicates within the same experiment. (F) PFCs were incubated with BCG and *M*.*tb*-related Ags for 8 hours. The cells were harvested and stained for the expression of IL-22 and IFN-γ by FCM. The representative dot plots were shown from three separate experiments with similar results. (G) Statistical results of IL-22 and IFN-γ expression on NK cells from PFCs. Data were expressed in box plots as medians, minimum and maximum values and statistical significance was determined with the Student’s t-test, *P<0.05 or **P<0.01.

Our previous study indicated that NK cells from PFCs produced significantly higher levels of IFN-γ under the stimulation with BCG compared to PBMCs. Therefore, we further characterized the production of other cytokines both by PFCs and PBMCs in response to BCG. The results showed that NK cells from PFCs but not from PBMCs expressed IL-22 in response to BCG. However, IL-17 was not produced by NK cells from PFCs or PBMCs under the stimulation with BCG (Fig 4A). Statistical results demonstrated that significantly higher level of IL-22 was expressed by NK cells from PFCs compared to NK cells from PBMCs (0.17%±0.06% vs 0.05%±0.009%, P<0.01) (Fig 4B). In addition, NK cells from PFCs could be divided into three distinct populations (Fig 4C). Further evaluation of cytokine expression showed that IL-22-producing NK cells did not co-expresse IFN-γ and IL-17 by NK cells from PFCs in response to BCG. These results demonstrated that a subset of the specific IL-22-producing NK cells was distinct from IFN-γ-producing NK cells in PFCs. Furthermore, we analyzed IL-22 and IFN-γ producing NK cells from PFCs in response to various *M*. *tb*-related antigens, including BCG, *M*. *tb*-HAg, *M*. *tb* -SoAg and *M*. *tb* -ScAg. As showed in the Fig 4D and 4E, addition of BCG, *M*. *tb* -HAg, *M*. *tb* -SoAg or *M*. *tb*-ScAg to cell cultures markedly induced the expression of IL-22 and IFN-γ by NK cells from PFCs. The statistical results indicated that significantly higher levels of IL-22 expression by NK cells from PFCs were induced with different TB antigens compared with medium (Fig 4E). Moreover, IL-12 and IL-15 significantly enhanced the expression of IL-22 and IFN-γ by NK cells from PFCs induced by TB antigens (Fig 4F). Most cells expressed either IL-22 or IFN-γ alone, and very few cells expressed both IL-22 and IFN-γ. The statistical results of three subpopulations of cytokine expression from five independent experiments were showed (Fig 4G).

### CD45RO^+^ memory-like human NK cells but not CD45RO^-^ NK cells from PFCs expressed and produced IL-22 in response to BCG

**Fig 5:**
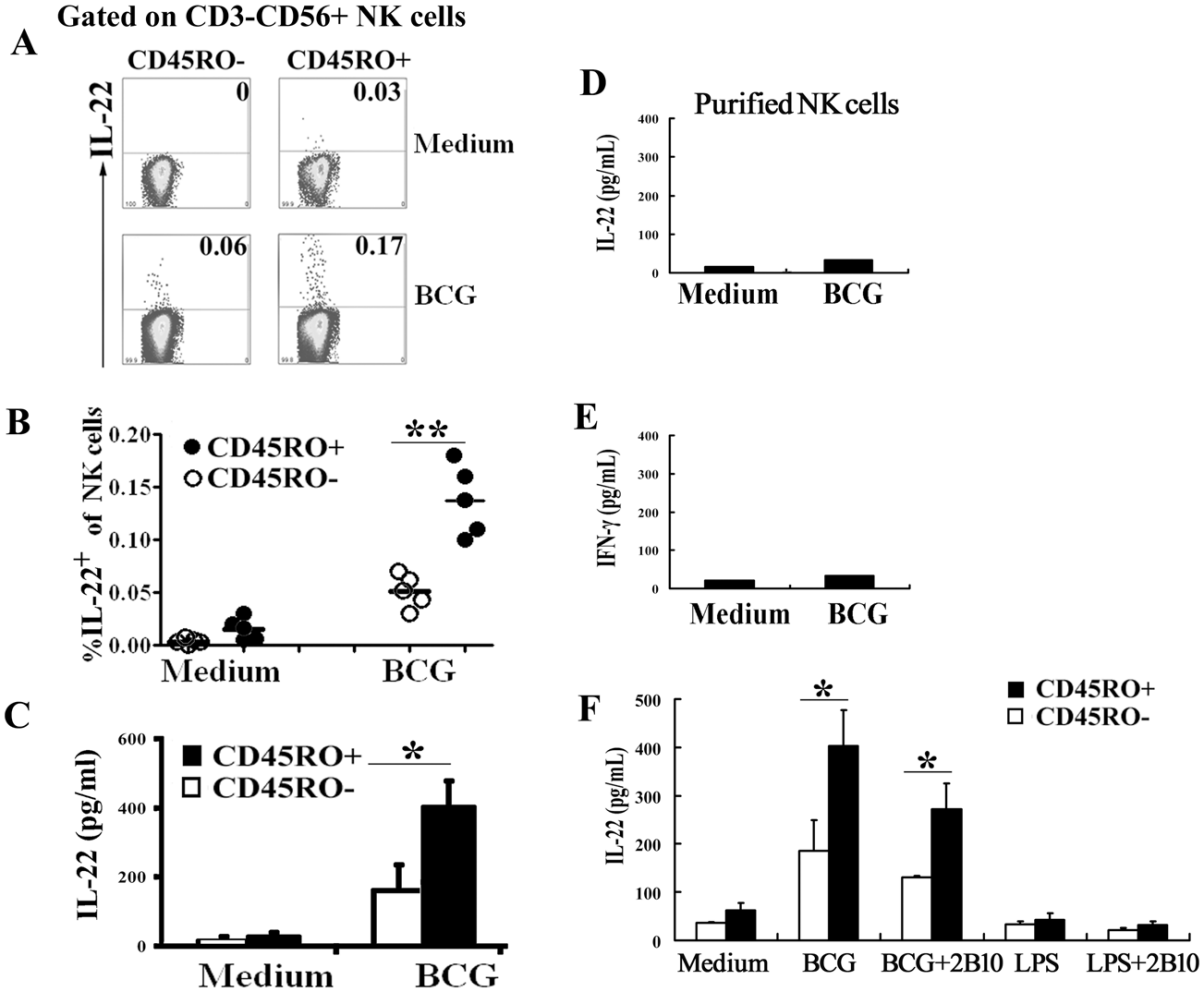
CD45RO^+^ memory-like NK cells from PFCs produced and expressed more IL-22 and IFN-γ in response to BCG. (A) PFCs were in culture with or without BCG and the expression of IL-22 by CD45RO^+^ or CD45RO^-^ NK cells was evaluated by FCM. Representative dot plots from five independent experiments were shown with similar results. (B) Statistical results of IL-22 expression by CD45RO^+^ or CD45RO^-^ NK cells from PFCs. The median and individual frequencies for each patient were performed. (C) Sorted CD45RO^+^ and CD45RO^-^ NK cells from PFCs were co-cultured with autologous monocytes at a ratio of 4: 1 in the presence or absence of BCG. The concentration of IL-22 was detected by ELISA. Statistical results were shown as mean±SD in histogram and error bars represent triplicates within the similar experiment. (D) and (E) Purified NK cells from PFCs were stimulated with or without BCG. The levels of IL-22 (D) and IFN-γ (E) were detected by ELISA. Statistical results were shown as mean±SD in histogram and error bars represent triplicates within the similar experiment. (F) Sorted CD45RO^+^ or CD45RO^-^ NK cells from PFCs were co-cultured with autologous monocytes at a ratio of 4: 1 with expected conditions. The levels of IL-22 (F) were detected by ELISA. Statistical results were shown as mean±SD in histogram and error bars represent triplicates within the similar experiment. *P<0.05 or **P<0.01.

To further gain the insight of cytokine production by CD45RO^+^ memory-like NK cells from PFCs, we evaluated the induction of IL-22 by CD45RO^+^ and CD45RO^-^ NK cells from PFCs. Purified NK cells were gated on CD3^-^CD56^+^CD45RO^-^ (left side) and CD3^-^CD56^+^CD45RO^+^ cells (right side). As shown in Fig 5A, CD45RO^+^ NK cells expressed significantly higher percentages of IL-22 (about two-fold to three-fold up) compared to CD45RO^-^ NK cells from PFCs after stimulation with BCG (P<0.05, Fig 5B). To confirm this result, we sorted CD45RO^+^ or CD45RO^-^ NK cells and were co-cultured with isolated autologous CD14^+^ cells (as antigen-presenting cells) from PFCs in the presence or absence of BCG. Cell-free culture supernatants were harvested at 48 h for the detection of IL-22 levels by ELISA. The results showed that significant higher levels of IL-22 were produced by CD45RO^+^ NK cells than CD45RO^-^ NK cells from PFCs, which was accordance with the FACS results (Fig 5C). However, purified NK cells from PFCs did not produce IL-22 and IFN-γ after stimulation with BCG (Fig 5D and 5E). These results strongly suggested that the production of IL-22 and IFN-γ by CD45RO^**+**^ NK cells was dependent on the presence of APCs.

Having confirmed that IL-15 and IL-12 could induce a stronger response for the expression of cytokines on CD45RO^+^ NK cells compared to CD45RO^-^ NK cells from PFCs, we further explored whether APCs, which secreted cytokines, displayed a critical role between NK cells and BCG. We isolated autologous CD14^+^ cells from PFCs as APCs and co-cultured with sorted CD45RO^+^ or CD45RO^-^ NK cells under the stimulation with BCG, LPS and antibodies against IL-12 receptors. As illustrated in Fig 5F, significantly higher levels of IL-22 were produced by CD45RO^+^ NK cells induced with BCG compared with those on CD45RO^-^ NK cells from PFCs. Furthermore, anti-IL-12Rβ1 mAbs (2B10) effectively inhibited the production of IFN-γ by NK cells (S2 Fig) but could not completely inhibit the production of IL-22 (Fig 5F). Simultaneously, LPS could induce the production of IL-12 but had no effect on the production of IL-22 and IFN-γ by CD45RO^+^ or CD45RO^-^ NK cells from PFCs.

### Cytokines and BCG enhanced expression of NKG2D, CD69, CD25 and granzyme B on IL-22-producing NK cells from PFCs

**Fig 6:**
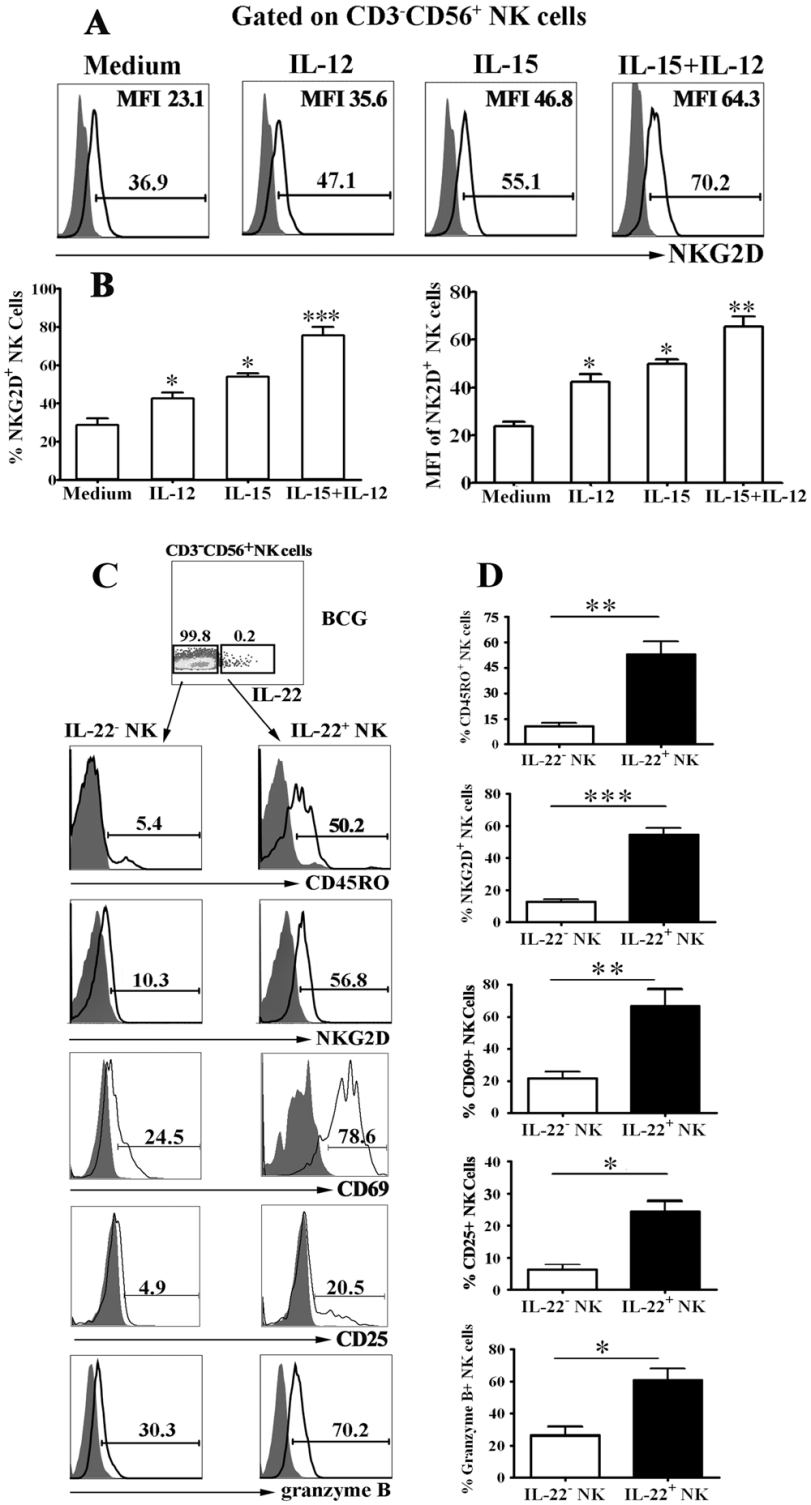
IL-15, IL-12 and BCG enhanced the expression of NKG2D and granzyme B by IL-22^+^ NK cells from PFCs. (A) PFCs were incubated with or without IL-15, IL-12 or IL-15 plus IL-12 and the cells were collected for the detection of NKG2D expression on CD3^-^CD56^+^ NK cells from PFCs by FCM. The representative histogram of NKG2D expression (open histogram) and isotype control (filled histogram) on CD3^-^CD56^+^ NK cells. (B) Statistical results were shown for NKG2D percentage and MFI on NK cells (n = 6). (C) PFCs were stimulated with BCG for 8 hours in the presence of BFA and the cells were harvested for the detection and characterizytion of IL-22 on NK cells by FCM. The cells were gated on CD3^-^CD56^+^IL-22^-^ (left side) and CD3^-^CD56^+^IL-22^+^ (right side) cells. The representative histogram of the phenotypes of NK cells were shown from three separate experiments with similar results. (D) Mean values of the results were shown as Mean±SD). Student’s t-test was used for statistical analysis. *P<0.05, **P<0.01 and ***P<0.001.

To investigate the possible mechanism of the response by CD45RO^**+**^ NK cells from PFCs under the stimulation with BCG or cytokines, we evaluated the expression of NK cell activation molecule-NKG2D. The results in Fig 6A and 6B demonstrated that IL-15, IL-12 or IL-15 plus IL-12 stimulation significantly enhanced the expression of NKG2D on NK cells compared with medium. Consistently, the MFI of NKG2D on NK cells was significantly increased in response to IL-15, IL-12 or IL-15 plus IL-12 compared with medium (Fig 6B). To further evaluate the phenotype of BCG-specific NK cells from PFCs on the basis of their ability to produce IL-22, we analyzed the expression of CD45RO as a marker for memory, NKG2D as NK cell activation molecule, granzyme B as a cytotoxic-related marker, CD69 and CD25 as activation molecules on IL-22-producing NK cells from PFCs after stimulation with BCG. As shown in Fig 6C, very low level of CD45RO was observed on IL-22^-^ NK cells but approximately 50% IL-22^+^ NK cells expressed CD45RO. Consistently, statistical result demonstrated that significantly enhanced expression of NKG2D, CD69, CD25 and granzyme B was detected on IL-22^+^ NK cells compared with IL-22^-^ NK cells (Fig 6D).

## Discussion

Immunological memory has been traditionally regarded as a unique characteristic of the adaptive immune response by T and B lymphocytes [33]. Memory cells are long-lived, respond more robustly to secondary infection, which are phenotypically and epigenetically distinct from their naive counterparts [34]. On the contrary, all other hematopoietic cells including natural killer (NK) cells are identified as innate immune cells, which have been considered short-lived but can respond rapidly against pathogens in a manner not though to be driven by antigen. Recently, several lines of evidence have established that NK cells can also be accessed by vaccination to deliver antigen-specific memory responses and protect against subsequent viral infections, which challenged the traditional distinctions between innate and adaptive immunity [15, 35]. A novel concept for which the term *trained immunity* has been proposed [36, 37]. Several mechanisms are involved in mediating innate immune memory, among which epigenetic histone modifications and modulation of recognition receptors on the surface of innate immune cells are likely to display a central role [38].

NK cells can kill autologous infected cells without prior sensitization, which mediate protection against viruses, bacteria and parasites. Human NK cells are commonly sub-divided into a CD56^+^CD16^-^ and CD56^±^CD16^+^ subpopulation. CD56^+^CD16^-^ NK cells play an important role as immune regulatory cells, bridging the innate and adaptive immune responses as they produce soluble factors including cytokines and chemokines. CD56^±^CD16^+^ NK cells are highly cytotoxic with very levels of cytokine production. Our previous data demonstrated that there existed memory-like human CD45RO^+^ NK cells in PFCs, which migrated to tuberculous pleural fluid via the IP-10/CXCR3 and SDF-1/CXCR4 axis. CD45RO^+^ NK cells in PFCs produce significantly higher levels of IFN-γ compared to CD45RO^-^ NK cells in response to BCG [17]. Yet little information was available with respect to the cytokine secretion of human memory-like NK cells except IFN-γ. In the current study, we further investigated whether human memory-like NK cells could contribute to immune defense through the production of IL-22. Considering that no pleural fluid was existed in healthy donors, PBMCs from healthy donors were used as controls to compare with NK cells from PFCs. Innate lymphoid cells (ILCs) are important innate immune cells, which participate in the transition from innate to adaptive immunity [39, 40]. NK cells are conventional cytotoxic ILCs. To identify NK cells from cytokine-producing helper-like ILCs (ILC1s, ILC2s, and ILC3s), the expression of surface markers (CD127, CD103, and NKp44) and transcription factors (Eomes, T-bet, RORγt, and Gata-3) should be characterized. However, it is hard to isolate live Eomes^+^T-bet^+^RORγt^-^Gata-3^-^ NK cells from PBMCs or PFMCs. In our study, we used CD3, CD16, and CD56 to identify and divide NK cells into three subsets as previous studies [17, 32].

Early reports indicated that NK cells produce not only IFN-γ, but also IL-22, a member of the IL-10 family of cytokines that is produced by CD4^+^, CD8^+^ T and NK cells [20, 23]. Consistently, our data demonstrated that human NK cells could produce IL-22 in response to IL-15 plus IL-12, especially in the subpopulation of CD56^high^CD16^-^ NK cells. IL-22 was originally described as a production of activated T cells, particularly by Th17 cells. IL-22 has been intensified because it was found to be a critical mediator of early mucosal defense against Gram-negative bacteria that cause intestinal disease and pneumonia in mouse models. In humans with *M*. *tuberculosis* infection, recent studies have found that memory like CD4^+^ T cells produce IL-22 and high level of IL-22 were present in bronchoalveolar lavage fluid of tuberculosis patients compared with those from healthy donors [11, 41]. Furthermore, our result illustrated that increased production of IL-22 but not IL-17 was observed on NK cells from PFCs compared to NK cells from PBMCs in response to BCG and *M*.*tb*-related Ags. In accordance with our findings, some reports demonstrated that human NK cells produce IL-22 in response to *M*. *tuberculosis* and restrict the growth of *mycobacterium tuberculosis* in macrophages by enhancing phagolysosomal fusion [24]. Another report proved that human NK cells produced IL-22, which inhibits intracellular growth of *M*. *tuberculosis*. Further study provides evidence that NK1.1 (+) cells and IL-22 contributed to the efficacy of vaccination against microbial challenge in a mouse model of vaccination with BCG [42]. Taken together, we hypothesized that NK cells from PFCs produce not only IFN-γ but also IL-22, which display a critical role in fighting against *M*. *tuberculosis*.

Recently, an increasing amount of evidence has been accumulated on the adaptive features of NK cells. In a mouse model of contact hypersensitivity, it has been advocated that NK cells could retain a long-lived and antigen-specific adaptive immune response independent of B cells and T cells. Paust *et al*. extended these observations and showed that a subset of NK cells in the liver acquires antigen-specific memory to various haptens and viruses. This capacity depends on their expression of CXC-chemokine receptor 6 (CXCR6) [30]. Infection of mice with MCMV is a well-established model of NK cell-virus interaction and some reports have established a view of NK cell memory that is based on viral antigen-driven proliferation through specific ligand/receptor interaction, which generates a self-renewing, long-lived memory population with enhanced ability to respond to a secondary challenge [43]. Yokoyama and colleagues found that activation by cytokines alone leads to the generation of NK cells with memory-like properties [28, 29]. Similarly, our previous study proved that CD45RO^+^ memory-like human NK cells responded more strongly and robustly than CD45RO^-^ NK cells in response to IL-12 or BCG [32]. Furthermore, our current study indicated that significantly higher level of IL-22 was measured on CD45RO^+^ NK cells treated with BCG compared to those on CD45RO^-^ NK cells from PFCs.

It was known that the effect between NK cells and BCG needed indirect contact which was strictly dependent on the production of cytokines by monocytes [44]. Our data illustrated that little level of IL-22 was detected on purified NK cells from PFCs when stimulated with BCG. In accordance with our prior study, CD45RO^+^ NK cells co-cultured with autologous monocytes exerted stronger response by inducing more IL-22 as well as IFN-γ when exposed to BCG than CD45RO^-^ NK cells from PFCs. However, anti-IL-12Rβ1 mAbs (2B10) could partialy inhibit the production of IL-22 by NK cells induced by BCG, indicated that IL-22-producing CD45RO^**+**^ NK cells co-cultured with autologous monocytes in response to BCG was dependent on IL-12 and IL-15. Consistently, some studies demonstrated that IL-15 is essential for the development and function of NK cells, and signaling through the IL-15 receptor in NK cells requires the adaptor molecule, DAP10, indicating an additional role for the IL-15/DAP10 signaling pathway in combating infection by intracellular pathogens [24]. More importantly, sorted NK cells from the patients with tuberculous pleurisy stimulated with LPS did not produce IL-22 or IFN-γ, indicating that the response of NK cells to BCG was TB-antigen specific. Further evaluation of activation molecule-NKG2D on NK cells demonstrated that IL-12 plus IL-15 synergistically induced higher frequency of NKG2D expression than medium. In agreement with our findings, some studies illustrated that the modulation of recognition receptors on the surface of NK cells may play a central role on the generation of memory NK cells [45, 46, 47]. The researchers clearly described the epigenetic characteristics of human adaptive NK cells in response to CMV [46, 47]. Simultaneously, IL-22 was produced preferentially by CD45RO^high^NKG2D^high^ granzyme B^high^CD69^high^CD25^high^ NK cells.

In conclusion, our experiments clearly demonstrated that the subpopulation of CD45RO^+^ memory-like human NK cells in PFCs express and produce IL-22 in response to BCG and TB-related antigens. We are aware of the present study being limited by a shortage of other groups to verify the epigenetic adaptation of memory-like NK cells. Despite its preliminary characters, our study demonstrated that CD45RO^+^ memory-like human NK cells in PFCs is an important component of host defense, and its discovery heralds a new step in our understanding of the adaptive features of NK cells. Nevertheless, the study of memory-like human NK cells is just getting started and our preliminary study will supply a novel sight for the evaluation of memory-like NK cells from TB patients.

## Supporting Information

S1 Fig IL-15 and IL-12 differently induce the production of IFN-γ in a dose- and time-dependent manner by human PBMCs. (DOC)

S2 Fig CD45RO+ memory-like NK cells from PFCs produced and expressed more IFN-γ in response to BCG which was dependent of IL-15. (DOC)

## Abbreviations

BCG: Bacille Calmette Guerin
IL-22: Interleukin-22
IL-15: Interleukin-15
*M.tb*: *Mycobacterium tuberculosis*
PFCs: pleural fluid cells
TP: tuberculous pleuritis
