# Supplementary material for: Memory-Like Antigen-Specific Human NK Cells from TB Pleural Fluids Produced IL-22 in Response to IL-15 or Mycobacterium tuberculosis Antigens
Source: PLoS One. 2016 Mar 31;11(3):e0151721. doi: 10.1371/journal.pone.0151721 (PMC4816314; doi:10.1371/journal.pone.0151721)
Supplement: S1 Fig — (DOC) [file pone.0151721.s001.doc]

**S1 Fig**

**
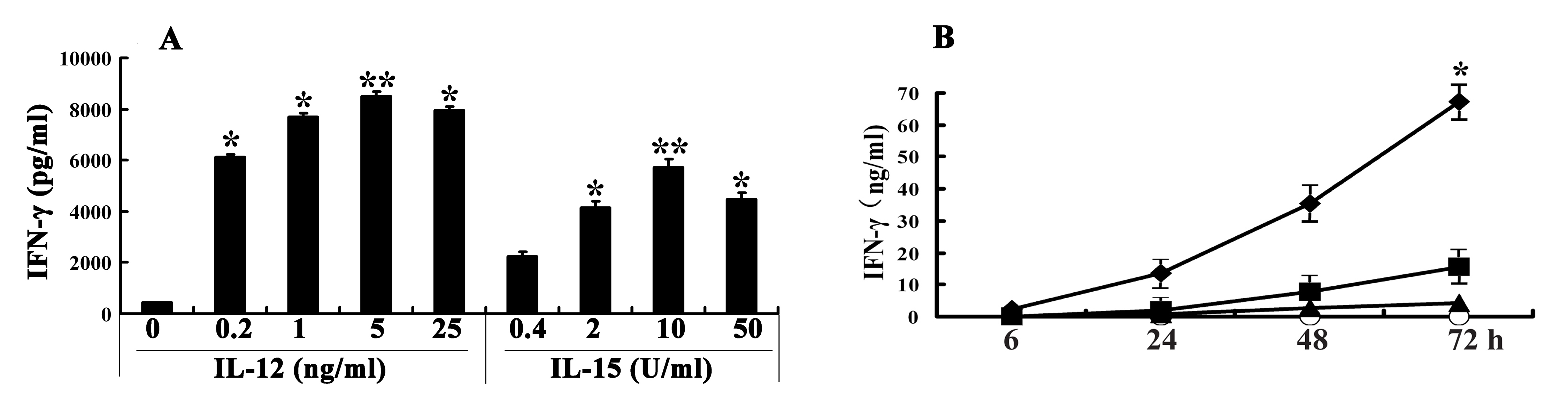
**

**IL-15 and IL-12 differently induce the production of** **IFN-γ** **in a dose- and time-dependent manner by human PBMCs.** PBMCs were incubated with or without IL-12 or IL-15 in different doses. The concentrations of IFN-γ (A) in the culture supernatants were determined by ELISA. Mean values of IFN-γ (n=5) were shown as mean±SD. In addition, PBMCs were incubated with or without IL-12 or IL-15 or IL-12 plus IL-15. The cells were harvested at different time points. The concentration of IFN-γ (B) in the culture supernatants were determined by ELISA. Mean values of IFN-γ (n=5) were shown as Mean±SD. Student’s t-test was used for statistical analysis, *P<0.05 and **P<0.01.
