# Supplementary material for: Memory-Like Antigen-Specific Human NK Cells from TB Pleural Fluids Produced IL-22 in Response to IL-15 or Mycobacterium tuberculosis Antigens
Source: PLoS One. 2016 Mar 31;11(3):e0151721. doi: 10.1371/journal.pone.0151721 (PMC4816314; doi:10.1371/journal.pone.0151721)
Supplement: S2 Fig — (DOC) [file pone.0151721.s002.doc]

**S2 Fig**


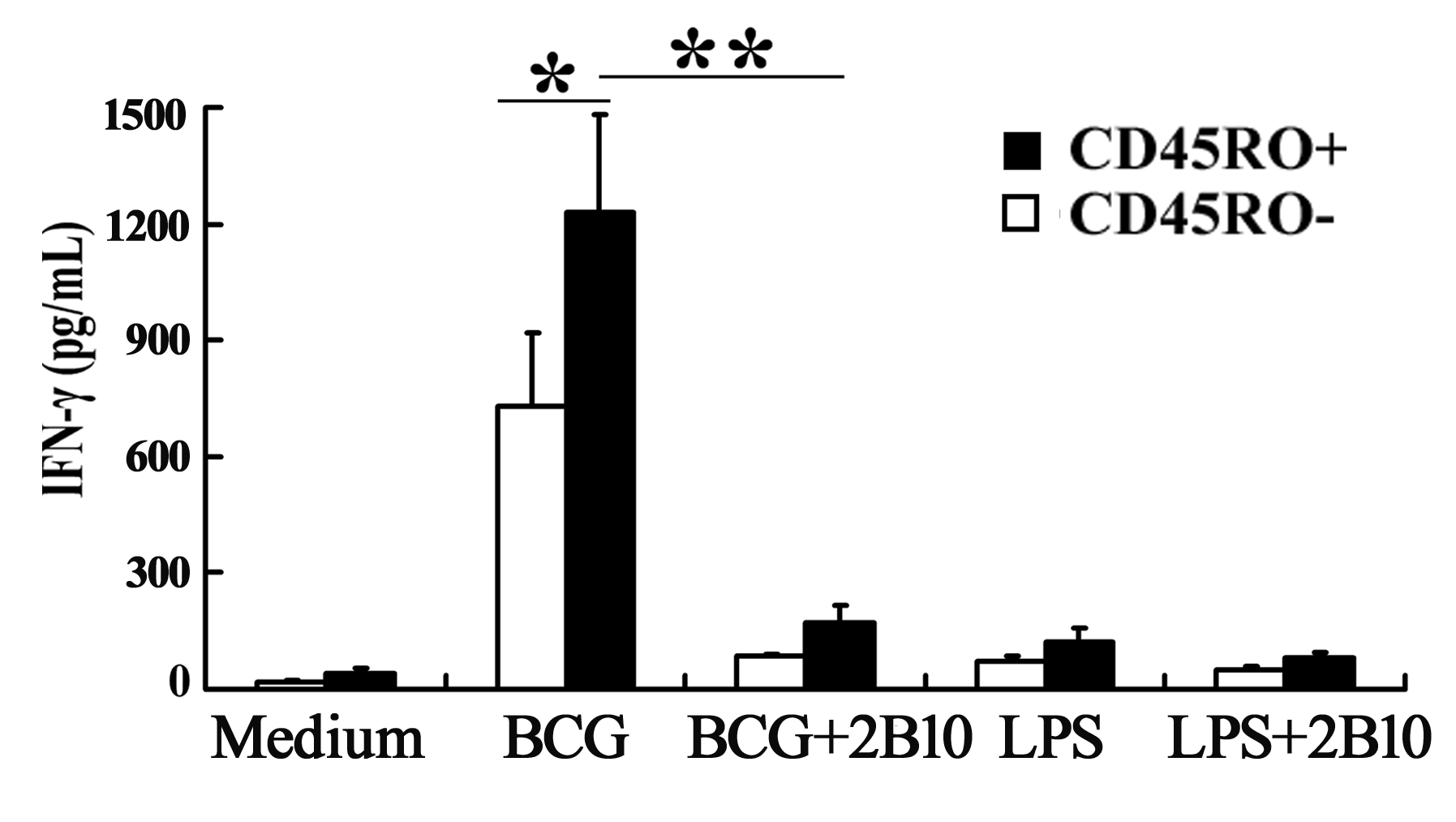


**CD45RO^+^ memory-like NK cells from PFCs produced and expressed more IFN-γ in response to BCG which was dependent of IL-15.** Sorted CD45RO^+^ or CD45RO^-^ NK cells from PFCs were co-cultured with autogenous monocytes at a ratio of 4: 1 with expected conditions. The levels of IFN-γ were detected by ELISA. Statistical results were shown as mean±SD in histogram and error bars represent triplicates within the similar experiment. *P<0.05 or **P<0.01.
